# The world is nuanced but pixelated: Autistic individuals’ perspective on HIPPEA

**DOI:** 10.1177/13623613231176714

**Published:** 2023-06-09

**Authors:** Greta Krasimirova Todorova, Rosalind Elizabeth Mcbean Hatton, Sarveen Sadique, Frank Earl Pollick

**Affiliations:** University of Glasgow, UK

**Keywords:** autism, HIPPEA, hybrid thematic analysis

## Abstract

Little attention has been given to the voice of autistic individuals during the development of theories that are trying to explain the condition. This can often make individuals feel that they have to fit into the theory’s definition, rather than it fitting into their experience. We aimed to understand to what extent the HIPPEA (High, Inflexible Precision of Prediction Errors in Autism) theory resonates with the lived experiences of autistic individuals. We conducted 21 questionnaires and 8 follow-up interviews and used a hybrid (deductive and inductive) approach to analyse the data. Based on the participants’ views, HIPPEA provides an explanation for many of the lived experiences of autistic individuals. However, refinement is needed with respect to interpersonal interactions, emotional processing and individuals’ motivation to engage with their environment despite challenges with the way the world is organised. Furthermore, more details are needed for the theory to accurately allow us to understand autism.

**Lay Abstract:**

Autism is a condition comprised of difficulties in social and communication contexts, sensory sensitivities as well as restrictive and repetitive behaviours. Many theories have tried to explain all the symptoms and behaviours associated with autism. We focus on one recent theory – High, Inflexible Precision of Prediction Errors in Autism (HIPPEA). We aim to understand how much this theory fits the experiences of autistic individuals. We collected data through 21 online questionnaires and 8 follow-up interviews. One of our participants was a parent of an autistic child, and the remaining were adults who reported a diagnosis of autism. We analysed the data by thinking about how it fitted with what we already knew and by looking for new insights which came up. Our results suggest that autistic individuals can make generalisations but that this happens more slowly across both social and non-social areas. These generalisations are very reliant on detail – in computer terms, they are ‘pixelated’. This is in line with what HIPPEA suggests. We also showed that autistic individuals can be motivated to explore and engage socially, something that needs more consideration within HIPPEA. Overall, this study shows that HIPPEA can explain many autistic experiences, but that further refinement is needed.

## Introduction

Autism Spectrum Condition (ASC) is multi-faceted with symptoms related to difficulties in social and communication contexts, a vast spectrum of hyper and hypo-sensitivities, restrictive and repetitive behaviours along with executive functioning difficulties and more (American Psychiatric Association, 2013). New theories have tried to provide a combined explanation of all symptoms and behaviours associated with autism by incorporating existing literature into a predictive coding framework (Lawson et al., 2014; Palmer et al., 2017; Pellicano & Burr, 2012; Van de Cruys et al., 2014, 2017).

Predictive coding postulates that the human brain is consistently anticipating the encounters of the world and checking these expectations (priors/predictions) against incoming sensory input (Friston, 2010). If there is a mismatch between the prediction and sensory input, then that forms a prediction error, which is propagated up the neural hierarchy. Whether or not the prediction error is taken into account depends on how precise the expectation is in specific cases. With high precision, more prediction errors are taken into account and with low precision, more prediction errors are disregarded as noise. The predictive coding perspectives of autism suggest that there is an imbalance between the precision given to predictions and the weight given to the sensory input. Pellicano and Burr’s (2012) theory proposes that individuals with autism form flat priors, suggesting that every new encounter is surprising. Conversely, theories such as Van de Cruys et al. (2014, 2017) and Lawson et al. (2014; Palmer et al., 2017) propose that there is a disproportionate level of precision given to sensory input which is consistently overestimated, making prediction errors highly relevant, even when they are the outcome of a noisy environment. The theories of Van de Cruys et al. (2014, 2017) and Lawson et al. (2014; Palmer et al., 2017) differ only in the emphasis of the type of uncertainty that needs to be estimated – the former highlighting inflexible, higher weighting of prediction errors by default, and the latter focusing specifically on the overestimation of the volatility of the environment as the cause of less flexible weighting of prediction errors. Nevertheless, both perspectives highlight that there is experimental support for a disproportionate level of precision given to sensory input which is consistently overestimated, making prediction errors highly relevant.

However, the extent to which these theories resonate with autistic individuals still needs to be established. To the best of our knowledge, since the enhanced perceptual functioning theory, which was developed in partnership with an autistic individual (Chown et al., 2017), the opinion of autistic individuals has not been sought on the other theories. The desire for inclusion in research is voiced by individuals and organisations, with one autistic individual commenting that they felt like they were expected to try and fit with the theories rather than the theories fitting them (Fletcher-Watson & Happe, 2019). Although theoretical frameworks provide much value in the advancement of the understanding of a condition, the extent to which a theory resonates with the individuals, whose lives it is trying to describe, is an important consideration.

Lived experiences are best explored qualitatively as this enables us to understand how individuals perceive the world (Lincoln, 2005). Using deductive qualitative analysis, which structures the research around a phenomenon (Gilgun, 2015), we can provide concept-guided descriptive research that is grounded in data. This gives a starting point to explore what people with autism think in terms of the processes underlying autism. Since much attention is being paid to predictive coding frameworks in autism, it is important to understand whether these theories resonate with individuals. This article focuses upon one particular theory – High Inflexible Precision of Prediction Errors in Autism (HIPPEA, Van de Cruys et al., 2014, 2017), which argues for inflexible precision associated with prediction errors in autism. We aim to understand the extent to which this theoretical account resonates with autistic individuals.

## Methods

### Ethics approval

Ethics approval was obtained for ethics application 300200118 through the University of Glasgow College of Science and Engineering Ethics Committee.

### Reflexivity

While we are attempting to provide an explanation of autistic individuals’ experiences, we do not identify as autistic. All authors have previous experience in qualitative research. The first author’s doctoral thesis focused on testing the validity of the HIPPEA theory (Todorova, 2021), which gave them a more theoretical understanding. Their experience of sharing their understanding of HIPPEA with autistic individuals prompted the current investigation into its relation to autistic experiences. Thus, they enter the study with the pre-conceived notion that the theory resonates with at least some individuals. At the time of data collection and analysis, the second author was an assistant psychologist, who worked with autistic individuals, both diagnostically and clinically. The third author was a psychology undergraduate student and came to the literature more naïve than the other authors. The fourth author is a senior academic and was the doctoral supervisor to the first author, indicating that they have similar pre-conceptions about the theory. They also have long-term experience in conducting research with autistic adults.

### Participants and recruitment

Recruitment was guided by theoretical sufficiency (Dey, 1999). Namely, the authors ended data collection when they felt the data described the deductively identified themes sufficiently.

Initially, 21 individuals participated in an online questionnaire. Participants were recruited through advertisement on social media, online forums and personal contact. To participate, individuals were required to confirm that they have a diagnosis from a clinician and are 18 or above or were parents of a child between 12 and 18 who had an autism diagnosis. Twenty participants were autistic adults, and one was a parent of an autistic child. We aimed to represent the views of both parents with autistic children and autistic adults; however, as there was only one parent, this study does not represent the views of parents of autistic children. However, the views of the parent did not differ from the other participants and therefore did not change the analysis or any interpretation of the results. Thus, to preserve the richness of the data, their data were not removed.

A miscommunication of the instructions occurred with three participants and therefore their data were removed. Specific data on race/ethnicity, socioeconomic status and educational attainment levels were not collected. The questionnaire was live between Winter 2020 and Spring 2021. Participant characteristics can be seen in Table 1.

**Table 1.** Participant characteristics for questionnaires.

N	Adult/child age (SD)	Gender M/F/NB	Age of diagnosis	Additional diagnoses^a^
21	33.95 (11.79)/13 (n = 1)	5/11/5	24.83 (14.04)	Anxiety (n = 6), Depression (7), Tourette’s (1), ADHD (3), PTSD (2), Dyspraxia (3), Dyslexia (1)

SD: standard deviation; ADHD: attention-deficit hyperactivity disorder; PTSD: post-traumatic stress disorder.

a These include mentions of being medicated for the condition or self-diagnosis or comments about previous diagnoses.

Interviews were then conducted during Spring/Summer 2021 with participants who agreed to take part in an interview in the questionnaire (n = 8, age = 35.75, SD = 15.22; age of diagnosis = 30.13, SD = 19.03; 5 female, 3 male, 1 female parent).

### Questionnaire development

A coding plan was first developed, guided by Van de Cruys et al. (2014, 2017). This ensured the questions solicited responses relevant to HIPPEA. The themes and the initial code books were developed separately by the first three authors who then converged to create one set. The initial deductive themes and the initial code book can be found in Supplemental Table A1.

The final questionnaire consisted mostly of open-ended questions (See Supplemental Information B). It started with questions on diagnosis status, additional diagnoses, gender and age. Next, participants’ general understanding of autism, autism theories and how they perceived their ability to understand themselves was queried. Participants were also given scenarios which tapped into the deductive themes mentioned above. Then, participants were introduced to a lay-person description of HIPPEA, written by the first three authors. Afterwards, they were presented with four vignettes describing autistic individuals and emphasising a specific part of the autism phenotype – sensory sensitivity, restrictive and repetitive behaviours, social situations, and stimming. Within these vignettes, we gave an example of how the theory would explain the portrayed behaviour and asked participants to provide examples from their lives that could be interpreted similarly. Finally, we asked participants for their overall comments about HIPPEA.

It should be noted that unfortunately neither the vignettes nor the lay summary were checked with the originator of HIPPEA. Therefore, the participants were responding to our reproduction of HIPPEA and not to the original theory.

### Community involvement

Three adults with autism (two male, one female) reviewed the final questions for clarity, relevance, ease of response and length. Several changes were made to the complexity and clarity of the questions; some were removed due to repetitiveness, one was added and some clarifications were made to the vignettes. One individual suggested that the questionnaire be sent to parents, which was added as a part of the study. Unfortunately, only one parent ultimately participated and therefore this remains an area requiring further work. The final questionnaire can be seen in the Supplementary Information B.

### Interview structure

A semi-structured interview schedule was developed after the analysis of all questionnaire data. Interview questions aimed to explore the themes identified from the questionnaire analysis. These themes focused on allocation of attention, complexity of the environment leading to uncertainty, exercise of control and agency, motivation and an overall theme focusing on what an autism theory should consider. The interviews also contained a longer description of the theory.

### Procedure

The surveys were self-administered using Qualtrics (https://www.qualtrics.com). An information sheet was first presented and consent obtained. At the end, participants were asked to provide their email if they wished to participate in a follow-on virtual interview. Participants were debriefed and contact information of relevant support services was provided.

For the interviews, participants were contacted to confirm their consent. They were then sent the interview questions with information that further questions may be asked. Interviews were held over MSTeams at a time convenient for the participant and were audio recorded. They varied in length between 1 and 2.5 h. The pace of the interviews was dictated by the participant. Participants were interviewed by the first and second authors.

### Analysis

This study employed a deductive qualitative approach which can provide concept-guided descriptive research that is grounded in data (Gilgun, 2015). Data were analysed using a hybrid approach intertwining theoretically driven thematic analysis and inductive engagement (Ashmore et al., 2017; Braun & Clarke, 2021). The adoption of this approach allowed themes to change, and new themes to be added. Themes were identified at a semantic level (Braun & Clarke, 2006) and a critical realist/contextualist perspective (Braun & Clarke, 2012) was taken. Data analysis was iterative, performed throughout data collection by the first three authors. The guidelines from Braun and Clarke (2012) through the six phases of thematic analysis were followed.

In phase 1, the authors familiarised themselves with the data and initial observations about the responses were noted. Where questions were based on an already provided example and the individual needed to indicate their confidence in understanding an example of the theory (see Supplementary Information B), the answers directly related to these questions were reviewed in the context of the participant’s confidence rating. Overall, participants reported high confidence in their understanding of the theory description and vignettes.

In phase 2, two steps were performed concurrently. The deductive codes were applied to the data and new inductive codes were identified. At this phase, an initial rigour check was performed. The first three datasets were double coded. These were then compared, and differences discussed. Definitions of codes were kept malleable to allow for expansion/separation.

In phase 3, the deductively identified themes were used to combine the originally identified codes along with others that fit the original theme description. Reorganisation of the codes and themes was done until themes provided a unique and standalone meaningful statement, grounded in repetitive though variant expressions (Herzog et al., 2019).

Phase 4 was a recursive process. It was not feasible to start and stop data collection to evaluate data saturation. Instead, after the first three cases were coded, the first three steps were repeated with every new 10 questionnaire responses, but the responses were split between the first three authors. For every 10 responses, one was cross-coded. New codes were discussed and added to the codebook. Themes were developed throughout and were evaluated against the whole dataset for meaningfulness. When no substantially different new codes were identified and the codes were clustering around coherent themes, data collection was stopped. Any additional responses collected in the meantime were analysed. The themes were reviewed to ensure that they fit with the data by considering the initial codes and impressions of the data.

In phase 5, the uniqueness of each theme was judged along with its relation to the research question. Themes were named, and appropriate extracts were chosen to sufficiently illustrate the theme and research question. Phase 6, writing of the analysis, was done concurrently with phase 5.

The analysis of the interview data followed the steps described above; however, coding and data collection were not done concurrently. The coding of the interviews was split between the first three authors, and one interview was triple coded. One adult participant’s interview data was lost as the recording failed. The interviewer noted down what they remembered, and it was checked to make sure the views were represented across the other interviews. New codes were incorporated into the revised themes from the questionnaire analysis. The meaningfulness of the outlined themes and the relationship between them was then adjusted based on the interviews.

## Results

### ‘It looks really nuanced but it’s pixelated’

HIPPEA argues that the development of generalisable concepts can happen in autism. However, it happens more slowly and is dependent on multiple experiences, allowing participants to create generalisable concepts that are based on much detail, rather than on abstract expectations. This makes the development of generalisable concepts more difficult.

In line with this, participants spoke about how generalisability can be difficult, with many referring to difficulties comparing one situation to another or having to consciously recall past experience to guide them because the situations were too different for this to happen naturally. This seemed to be the case for both social and non-social situations:

–we experience situations that are only marginally different from a previous one, as completely new, because . . . it technically *is*. Every time I meet a dishwasher, I need help operating it, even though I *know* that a given sequence of actions *probably* means my dishes will be dry at the end. (Questionnaire 2)^1^

However, this was not the case for everyone:

. . . social situations are often similar for me despite surface differences. (Questionnaire 9)

However, if there was a lot of information available, either through them consciously attending to more details in the moment or through previous experience, participants indicated that they could create a more generalisable understanding of their environment with time. This led them to be more confident but still cautious:

. . . I am someone that has really . . . from a really young age umm thought a lot about why people are doing what they’re doing and so . . . my social map . . . it looks really nuanced but it’s pixelated. (Interview 4)

This was not the case for all. Some participants would more naturally recall previous experiences instead of consciously recalling them to provide a basis for comparison. The differences between previous experiences being drawn on naturally or through cognitive effort may depend on how similar and recent situations are:

If it was like a small comparison it probably happen very similar, whereas if I’ve had a time where I have actively had to think about it, it has either been a long time since it has been and so I have tried to think back maybe 2/3 years ago vs like this is similar to this but they are both still different . . . (Interview 5)

#### Expectations based on emotional experiences

In the questionnaires, participants described how strong emotional experiences lead to the development of expectations about certain events or occurrences happening in specific ways. This sometimes leads to anxiety about the possibility of the event reoccurring or to disappointment when the expected positive experience does not occur:

If I have visited a place that was unexpectedly very hot or cold for the type of place it is . . . I will plan for that occurrence to recur on any future returns to the same place, even if the initial conditions are unlikely to be in effect. Those circumstances will be memorable and I will treat them as probable, forever. (Questionnaire 4)

However, in the interviews, some participants were unsure if this would happen after a one-off event. Instead, it was suggested that a strong emotional response could tip the scales in a certain direction.

### ‘the more complex the situation the more you are processing’

As expected from the theory, the more complex the environment – social or non-social – the more difficult it becomes to navigate. This was usually caused by the disruption of routine, which then potentially required the person to develop a new expectation about what the new situation needed of them:

When something does disrupt my routine, it depends on what it is. If I can ignore it or if it’s a very slight disruption it’s alright. If it’s something I see as bigger, then I usually shut off until I’ve gotten over it. Like one morning my light didn’t work, which objectively is not a big deal, but it made me very anxious because that is THE first thing I do in the morning so I ended up getting out of bed much later because it made me very anxious. (Questionnaire 9)

However, it was suggested that, at least for some, once complexity in the environment exceeds a certain level, it has the same impact regardless of further complexities. Thus, there is a capacity for uncertainty that can be tolerated, which once surpassed requires the individual to prioritise dealing with one of the stimuli:

point of like complexity that environments get to that . . . The impact of that on me. It doesn’t matter how much complexity comes after that [. . .] if there’s a room with 12 people in it, that would have the same impact as a room of 200 people in it . . . It doesn’t become more complex after a certain point. (Interview 7)

While complexity decreased coping, familiarity, both socially and environmentally, was seen to be helpful, because it reduced the amount of new or unexpected elements that needed to be acted upon:

with friends, I know them enough to not be surprised by any chance in circumstance, and I will never be caught off guard in a social situation. (Questionnaire 18)

However, one participant shared how both the situation and the person would have to be the same for them to feel at ease:

. . . it tends to be for a particular person or a particular situation, if for example, I hit the same situation but with a different person, that I didn’t have the same depth of experience with then I would have the same difficulties as I had with the original person in the original situation right at the beginning. (Interview 1)

This familiarity with the environment was also related to growing up and learning more about how complex environments can be and how to deal with them. This ties in with the previous theme about generalisability and HIPPEA in general, where generalisability develops over time, with continuous exposure:

I think when I was younger, I used to get a lot more anxious about going into a new situation. I feel a lot more confident about that now as I have gotten older. (Interview 8)

#### Antecedents to coping abilities

The participants’ emotional state appeared to affect their ability to cope with the demands of the situation (social or not). Participants reported that if they were already feeling stressed or overwhelmed, then they would find it more difficult to use existing coping mechanisms or deal with uncertainties:

I think if I am very tired or if I am already upset about something or if, especially if I’ve had a bad social interaction, emm, then I am more likely to get overwhelmed about something else. (Interview 3)

The complexity of the environment and the difficulty of dealing with the world can be increased by society’s expectations. Societal norms tend not to allow for the fact that neurotypicals and autistics have ‘different operating system[s]’ (Interview 1). To help with this, some participants spoke about trying to be open, to help others understand how they interact differently with the environment. Being surrounded by understanding individuals was suggested to help lower the load:

I’ve been trying to be very open about being autistic in my academics and everything. . . . what I really am looking for is just kind of benefit of the doubt. If I am not making eye contact, I’m still listening to you or if I’m if I just respond in some way that’s really awkward. . .so that I can maybe be myself a little bit more. (Interview 8)

However, opening up to others about what it is like to be autistic can be difficult:

it’s just better than having to explain why you have done something that’s weird, emm, it’s worth the time if they are your friends or they are people who you feel comfortable with but if it’s just someone who is your colleague or something, it’s just not worth the emotional effort. (Interview 3)

#### Emotions and social situations

Our participants’ reflections showed that social interactions bring a different level of complexity:

. . . the kind of things people think are a normal level of engagement are just way more for me and they don’t understand it, I can’t explain that to everybody in every situation, even people who know cognitively what the issue is for you, don’t know, still find it really hard to understand. (Interview 4)

When discussing social interactions, it is paramount to also discuss the processing of emotions. To provide context, the interview participants were asked for their definition of empathy. Participants gave similar definitions that described being able to understand what the other is feeling and to feel the emotions they are going through. In this way, participants ended up describing themselves experiencing cognitive or emotional empathy to various degrees (Cuff et al., 2014). However, they also described difficulty dealing with others’ emotions, because it can be difficult to understand their own. In this sense, some participants described empathy more similarly to understanding the reason behind someone else’s feelings, whereas others described feeling others’ emotions sometimes to the extreme. One participant also expressed that they do not understand their own emotions but would still try to show understanding for those of others, saying they would ‘intellectually understand’ (Interview 8).

Those that spoke more about being able to feel the other person’s emotions also spoke about how if they are upset themselves, this can make the situation even more complex as they have to manage their own emotions as well as the other person’s:

. . . feeling really overwhelmed by especially if someone is really upset . . . I find it really hard to sort of stay centred myself and not to get caught up in it and then also I often find it hard to know what to do in response or what to say in response. (Interview 8)

Overall, however, participants tended to take a more analytical and rational perspective to interactions that required empathetical responses. Thus, whereas on the outside it might appear that the autistic individuals may not empathise because of that approach, it is not due to a lack of understanding:

but there’s been times where it’s been like, those people I work with, gymnasts . . . if they may react in the way I’ve reacted before or how I would react in a very similar situation but because I am on the outside of it, it is much easier to be like, this isn’t the best way to react. (Interview 5)

### Motivation

It was clear that despite the difficulties, our participants tended to search for ways in which to interact with the environment and people.

#### ‘Constantly masking with [. . .] friends, at work, with [. . .] family’

Participants often spoke about how they will disregard their preferred way of interacting with the environment. Although this was often done to the detriment of the individual, it was a deliberate decision to avoid isolation and negative consequences from others’ reactions and to avoid missing out on life:

. . . I think sometimes it’s worth putting up with certain things to emm, to be part of, to have that experience . . . sometimes it’s also about meeting the expectations of others, emm that was a big part of it when I was growing up. (Interview 8)

#### Specific interests bring satisfaction/accomplishment and calm

Specific interests helped participants explore, feel in control and construct their world. They also brought calmness. A few focused their special interest on understanding themselves or neurotypicals through studying Psychology/Sociology.

Computer games – they are very controllable and generally predictable. Even the unpredictable elements tend to follow a design philosophy that is recognisable after playing so many games. I also enjoy the feeling of being powerful and in control so tend to play single players game where you are almost always going to be the hero protagonist. (Questionnaire 3)

### Exercising control in an overwhelming environment

To cope with overwhelming environments, participants drew on both pro-active and reactive strategies, including planning, taking on a role in social situations, having a bath and using fidget toys. One participant spoke about how they will reduce their stimulation in preparation for an event that is going to happen. As mentioned earlier, however, the person’s emotional state can affect the strategy used:

Also, what I’m aware is going to happen in the next day or 2 after, plays a big part in what I expect. You know, like, how much of myself I’m willing to expend in energy . . . to that interaction, I do almost hold back sometimes, [. . .]. I could be taking part of, taking time with a friend and it’s a good environment and a good level of social interaction. But if I know that the next day I’m gonna have to do something very similar, I’m going to cut that shorter, than its maybe typically expected. (Interview 7)

### What an autism theory should consider

Some participants expressed their general frustration towards autism theories due to their negative portrayal of autism:

‘Refrigerator mother theory’ – this is an old theory that autism is caused by poor parenting. It has long since been discredited, but as an autistic parent I have still come into contact with people who believe this theory or some version of it. I believe this to be the reason that children are often removed from the care of autistic parents – it’s believed that our children are autistic because we do a poor job of parenting. (Questionnaire 17)

They described how the theories fail to capture both the differences within the autistic community and the world from an autistic perspective; wrongly suggesting that anything other than the neurotypical experience is detestable and ignoring the difficulties that neurotypicals also face when interacting with autistic individuals. Participants called for theories to consider the positive aspects of autism along with the difficulties:

attention to detail . . . is actually kind of incredible in some ways it just gets really . . . misapplied in social situations or, not that we can turn it off at will, obviously, right?, so that’s the problem you know, emm but there are all these hyper perceptions that are involved and that’s not always bad. (Interview 6)

In terms of HIPPEA, there were mixed feelings across participants about whether it made sense to them. While some felt like it explained their lived experiences well, others felt that the theory is still in its early stages. Nevertheless, participants seemed to agree that they processed information in more detail than neurotypicals which resulted in them spending more time deciphering it:

. . . like designing a programme, you have to account for different input and obviously if you don’t tell them, the program what input to expect, if you get unexpected input it tends to completely stop or it impacts on the output you get. (Interview 5)

However, the participants felt that HIPPEA was still too ‘deficit based’ (Interview 3) reminiscing of their experience of other theories.

## Discussion

Our aim was to explore whether HIPPEA resonates with autistic individuals. Following a hybrid deductive/inductive approach to analyse data from autistic individuals and one parent, we can see that whereas HIPPEA’s propositions resonate with some, there are differences between individual experiences and views on the theory.

Allocation of attention to small differences appears to be a common occurrence and generalisability is difficult across social and non-social domains. However, individuals are able to generalise albeit more slowly. This is a nominal point in HIPPEA – generalisability takes time but with continuous exposure, generalisable concepts will be formed (Van de Cruys et al., 2014). However, as one participant put it, these will look nuanced, but be very ‘pixelated’, reliant on much detail. Disentangling random changes from true/relevant environmental changes is more difficult, hence concepts being more pixelated.

One phenomenon we observed in relation to attention to detail was the emotional load of the event. In relation to avoidant and seeking behaviours, we observed that strong emotional experiences could tip the scale towards expecting the event’s repetition. Although it was evident that participants do not think negative experiences will always happen, they talked about there always being a possibility and thus wanting to avoid it. The enhanced weighting of negative events has also been suggested by a recent investigation into probable post-traumatic stress disorder and autism, where it was found that autistic individuals were more likely to experience post-traumatic stress symptoms (Haruvi-Lamdan et al., 2020). Haruvi-Lamdan et al. (2020) suggest that autism serves as a vulnerability for post-traumatic stress disorder. Similarly, links have been found in individuals with autism and depression, suggesting comorbidities with depression might lead to this negative attentional bias (Bergman et al., 2021). Thus, it is possible that participants’ exaggerated responses to negative experience and their subsequent avoidance might be an indication of such comorbidities. However, it is equally possible that such biases are based on increased negative experiences in autism compared to the neurotypical population (Griffiths et al., 2019; Pearson et al., 2020) or increase recency bias as proposed by the HIPPEA theory, where negative, but recent experiences bias autistic individuals into expecting more negative future experiences since they are weighted higher. A different interpretation of this finding can be around reduced optimism bias, whereby neurotypical populations have been found to be more biased towards positive beliefs about the future – congruent with smaller belief updates after encountering undesirable information (Eil & Rao, 2011; Garrett & Sharot, 2014; Kuzmanovic et al., 2016). There have been some indicative findings that individuals with high autistic traits experience less of this optimism bias and in turn are more realistic in their expectations – enhanced rationality (De Martino et al., 2008; Kuzmanovic et al., 2019; Rozenkrantz et al., 2021). Quantitative investigations into these possibilities, allowing for controlled disentanglement of causes, would be needed to identify the one that provides the best fit for the differences in experience expectations between autistic and neurotypicals.

The second theme we examined was around complexities in the environment. Participants indicated that interacting with the world can be difficult because of the many elements that need to be processed. Van de Cruys et al. (2014) explain that this is because the natural environment presents many open-ended and unpredictable circumstances. As highlighted by our participants, familiarity with the environment or individuals will positively influence the response to a situation as fewer new elements will need to be estimated. Familiarity, thus, engenders trust in an environment or a person helping to cope with vigilance caused by uncertainty (Murray et al., 2023). Internal states – that is, mood – additionally complicate one’s ability to deal with changes in one’s surroundings, which was evident here. To cope, participants would first try pro-active strategies but would then use reactive strategies if needed.

Inductive sub-themes on emotional processing show how complex social interactions can be. Whereas the initial HIPPEA articles do not explore empathy deeply, a subsequent paper that investigates interpersonal interactions suggests that social interactions and reciprocity require a person to attribute variability associated with the present situation and emotions appropriately between themselves and their partner (Constant et al., 2020). In a neurotypical interaction, an individual can appropriately attribute variability to the context and others’ mental states, thus responding accordingly, allowing space for others’ variable behaviour (Constant et al., 2020). Difficulties with attribution of uncertainty away from one’s own experience and towards more abstract concepts, like emotions, can make social interactions challenging. Such difficulties might be underlined by a difficulty in disentangling one’s own from others’ emotions. Nevertheless, our participants expressed that they can understand others’ emotions; some even described high levels of personal distress in response to others’ suffering. This has also been observed in quantitative studies suggesting that there might be an imbalance of cognitive and affective empathy in autism. Specifically, and in accordance with some of our participants’ accounts, autistic individuals have been shown to have heightened affective as opposed to cognitive empathy, with brain areas associated with empathic arousal showing heightened activation, but areas associated with social understanding of others’ distress showing reduced (Fan et al., 2014; Shalev et al., 2022). However, our participants spoke about how they may struggle knowing how to respond, with some choosing to offer more practical rather than emotional support. Emotions are complex regardless of whether they are our own or others’, and if an autistic individual is trying to cope with an already complex environment, it may be easiest to use a practical approach.

The authors of HIPPEA also suggest that often-associated problems with empathy might be in relation to alexithymia as proposed by R. Cook et al. (2013), and at least one participant indicated that they do not recognise their own emotions unless they reach the extremes. It is possible that most participants might have been those who do not exhibit alexithymia. Indeed, it has been suggested that once controlled for alexithymia, differences between autistics and neurotypicals in terms of empathy tend to disappear (Santiesteban et al., 2021). However, it is certain that all our participants described empathetic experiences – cognitive, affective (Cuff et al., 2014) or elements of both. Overall, when it comes to emotional responses and interpersonal interactions, HIPPEA might provide a general statement of explanation – that is, that the complexity of social and emotional interactions is too abstract and too variable to be easily learned. Although Constant et al. (2020) attempt to provide more detailed insights, more in-depth understanding of how interpersonal interactions fit within the HIPPEA model is needed.

A third theme was around motivation. According to HIPPEA, the difficulties with precision can lead to frustration and withdrawal, caused by difficulties with estimation of uncertainty. However, our participants were motivated to explore their environment, for example, through their special interests, which helped them feel calm and relaxed. This was also found in Dachez and Ndobo (2017). Van de Cruys et al. (2014) describe special interests as a way of introducing structure by engaging in repetitive behaviours and actions. In addition, some participants used their special interests, such as psychology or sociology, to understand the world, which was also raised by the autistic participants in Dachez and Ndobo (2017). HIPPEA argues that the transition from a high prediction error to low prediction error environment can provide positive affect and such rewards motivate autistic individuals to achieve this state potentially through the continuous engagement with an activity to find all its variations, thus reducing its unpredictability. Therefore, it can be assumed that engaging in specific interests could affect the amount of prediction errors by reducing them. It is similarly possible, within HIPPEA’s interpretation, to allow for an increased number of new experiences in a controlled environment, where only minute differences will be observed. However, little space is provided to the development of special interests in the HIPPEA theory, but there is potential within its current development to allow for the interpretations given in this article.

Participants were also motivated to engage socially. Sometimes this led to the use of masking, which manifested in both avoiding their own desired behavioural patterns to accommodate changes in the environment and masking their autistic characteristics, to facilitate their social interactions or avoid adverse reactions from others such as bullying. These reasons for masking have been previously reported (e.g. A. Cook et al., 2017; Sedgewick et al., 2021). This is despite other theories discussing a lack of social motivation in autism (Chevallier et al., 2012). Although HIPPEA suggests that autistic individuals will engage in withdrawal due to the unpredictability of social interactions, our findings and other arguments on masking do not support this.

On discussions around theoretical accounts of autism in general, our participants focused on how theories have traditionally framed the condition as deficit based and have ignored the many positive aspects of autism. Traditional autism theories (i.e. theory of mind, weak coherence theory and executive functions theory) reduce autism to its underlying neurobiological mechanism and struggle to explain the heterogeneity among autistic people (Happé et al., 2006; Waterhouse & Gillberg, 2014). The participants in our study rejected these theories due to their deficit approach. While some participants felt that the HIPPEA theory was more accommodating to their experiences, others felt it followed the same path as previous theories and took a deficit approach, not accounting for heterogeneity. This could have been due to how we described the theory. Nevertheless, it is important to ensure theories are not deficit focused (Evans, 2013), especially as the ‘deficits’ in autism may be because society does not accommodate an autistic person’s needs (Fletcher-Watson & Happe, 2019). For example, both autistic and non-autistic people have difficulty communicating with each other and it is not an impairment reserved to the former (Williams et al., 2021).

Bervoets and Hens (2020) specifically position HIPPEA as not being deficit based. Namely, HIPPEA describes how heterogeneity is to be expected (Constant et al., 2020; Van de Cruys et al., 2014) due to every autistic person using different resources from their environment as building blocks to construct their perception of the world. The priors they build are subjective to their experience, against which the prediction errors are produced, and the salience of prediction errors depend on their personal context (Bervoets et al., 2021). Similarly, Bervoets et al. (2021) expand on this by suggesting that the mechanisms are the same in the autistic and non-autistic individual, but the source of uncertainty is where the differences lie – autistic people are more sensitive to prediction errors, which means there is a high chance of them missing learning opportunities ultimately leading to increased levels of uncertainty (Palmer et al., 2017). Through these trajectories autistic individuals shape their environments more tightly (Bervoets et al., 2021), or as one participant referred to it – their worlds are ‘more pixelated’. When a world is designed by and for a neurotypically developing brain, interacting with it will lead to higher levels of uncertainty for an autistic individual (unlike the ones that they would build themselves such as the worlds built through special interests as suggested earlier in this discussion and as described in the monotropism theory; Bervoets et al., 2021; Murray, 2018). In reality, these more tightly defined worlds can lead to exceptional strengths in autism along with difficulties when navigating a world not built for a mind that puts high weight on prediction errors. In this frame, HIPPEA in itself is not to be seen as a deficit based theory, rather as representing an underlying mechanism – high and inflexible precision of prediction errors – which can encapsulate various autism phenotypes.

## Limitations

Our interpretations need to be taken in context with the limitations of the study. It may have been difficult for some autistic participants to reflect on their experiences. Due to the nature of introspection required, it is likely that all participants had strong cognitive skills, meaning that experiences of other groups of people with autism may not be represented. In addition, we were only able to recruit one parent meaning that further research is needed in order to gain a sense of the views of parents. Moreover, since we used online questionnaires, we were unable to gain clarifications or clarify misunderstandings. For example, one questionnaire participant was upset with how the theory had been simplified. The interviews aimed to improve on this by offering more information and space for discussion. Although we were only able to follow up with a small subset of participants, we used all the responses from the results to clarify any inconsistencies or potential misunderstandings. Finally, we did not confirm the accuracy of the layman reproduction of the theory with the originator of HIPPEA. Therefore, all responses are in response to our interpretation and reproduction of the theory. Although we tried to represent the theory well, it is possible that the participants would have felt differently if the originator of the theory had been able to explain it themselves. It is also important to acknowledge our realist/contextualists perspective and the nature of lived experiences as evidence to underlying mechanisms. Although we show that lived experiences largely match with the predictions made by HIPPEA, lived experiences cannot always provide us with direct insight into underlying mechanisms. Lived experiences are by nature interpretations of these mechanisms and cause and effect as well as compensatory reactions cannot easily be disentangled. Thus, what could be seen supporting or refuting a theory based on lived experiences could be misleading and quantitative evidence in complement of our qualitative results is needed to provide a cohesive understanding.

## Conclusion

We highlight that HIPPEA provides an explanation to most of the lived experiences of autistic individuals. The transition from fixed to generalisable concepts of social and non-social environments was reported to require more exposure due to the effort that goes into the reduction of prediction errors. Furthermore, the inherent complexities that exist in any environment increase the uncertainty for individuals which may result in negative experiences. These were found to be pronounced in social interactions, particularly, when empathising was required. Therefore, participants tended to favour routines and habits or engage in repetitive behaviours as these actions limited the exposure to more information and warranted certainty. However, these complexities did not stop our participants from exploring new environments, allowing them to engage in various special interests and create their own world. Sometimes, the motivation to interact with people encouraged them to mask their autistic traits despite the process being cognitively taxing. These concepts are vaguely addressed by HIPPEA and thus need more elaboration. Hence, there is room for refinement.

## Supplemental Material

sj-docx-1-aut-10.1177_13623613231176714 – Supplemental material for The world is nuanced but pixelated: Autistic individuals’ perspective on HIPPEA Click here for additional data file. Supplemental material, sj-docx-1-aut-10.1177_13623613231176714 for The world is nuanced but pixelated: Autistic individuals’ perspective on HIPPEA by Greta Krasimirova Todorova, Rosalind Elizabeth Mcbean Hatton, Sarveen Sadique and Frank Earl Pollick in Autism

sj-docx-2-aut-10.1177_13623613231176714 – Supplemental material for The world is nuanced but pixelated: Autistic individuals’ perspective on HIPPEA Click here for additional data file. Supplemental material, sj-docx-2-aut-10.1177_13623613231176714 for The world is nuanced but pixelated: Autistic individuals’ perspective on HIPPEA by Greta Krasimirova Todorova, Rosalind Elizabeth Mcbean Hatton, Sarveen Sadique and Frank Earl Pollick in Autism

## Notes

1. Quotes are reported from both the questionnaires and interviews, indicating where the quotes come from. The questionnaires and interviews are given arbitrary numbers, meaning that person associated with questionnaire 2 is not necessarily the same person as the one from interview 2.
