# Supplementary material for: The world is nuanced but pixelated: Autistic individuals’ perspective on HIPPEA
Source: Autism. 2023 Jun 9;28(2):498–509. doi: 10.1177/13623613231176714 (PMC10851622; doi:10.1177/13623613231176714)
Supplement: sj-docx-1-aut-10.1177_13623613231176714 – Supplemental material for The world is nuanced but pixelated: Autistic individuals’ perspective on HIPPEA [file sj-docx-1-aut-10.1177_13623613231176714.docx]

Supplementary Information A

The initial deductive codebook is presented below in Table A1.

Table A1. Initial code book.

| Allocate attention | Environment | Motivation | Control |
| --- | --- | --- | --- |
| An individual with autism will be less likely to consider variations in situations as noise, thus they create inflexible and concrete predictions; An individual will consider noise in the environment as being equally important to learnable variation leading to reduced generalisability | The more complex the environment is, the more difficult it becomes to estimate. | A constantly high level of precision brings about frustration and withdrawal in complex situations; strict and easily predictable situations lead to feelings of reward and consequently the reinforcement of that behaviour. | An individual will try to influence the stability of the environment by performing behaviours that produce expected outcomes |
| IF = Inflexible or concrete predictive/ learnt templates  UTP = Unable to tune precision | COMP = complexity  SCOMP = social complexity | MELR = Motivation to explore and learn to gain reward  (Extrinsic and Intrinsic) | DWUOE = Dealing with the uncertainty of the environment |
