# Supplementary material for: The world is nuanced but pixelated: Autistic individuals’ perspective on HIPPEA
Source: Autism. 2023 Jun 9;28(2):498–509. doi: 10.1177/13623613231176714 (PMC10851622; doi:10.1177/13623613231176714)
Supplement: sj-docx-2-aut-10.1177_13623613231176714 – Supplemental material for The world is nuanced but pixelated: Autistic individuals’ perspective on HIPPEA [file sj-docx-2-aut-10.1177_13623613231176714.docx]

# Supplementary Information B

The questionnaire that participants responded to can be found below.

1. You can have someone help you complete this questionnaire, like a friend, a parent, a carer. Do you have someone assisting you with answering the questions. If yes, please specify what is their relationship to you. (yes, no, conditional free on yes)

**Introduction**

1. What is your date of birth?
2. What is your gender? Male/Female/non-binary/ Prefer not to say
3. Is your gender the same as the one that you were assigned at birth? Yes/No/Prefer not to say
4. Have you been formally diagnosed by a professional psychiatrist/psychologist, that you have an autism diagnosis? (Yes/No/Self Diagnosed)
5. Do you know what you were specifically diagnosed with? If you do, please state it below (e.g ASC/ASD, Autism, Aspergers).
6. How old were you when you received this diagnosis?
7. Do you have any other mental health or neurodevelopmental diagnoses? (E.g. anxiety, depression, ADHD, language impediment)
8. How well do you think you understand yourself and your own experiences? *Sliding scale 1-10(not at all to Very much so)
9. Do you think you experience the world differently to others who do not have an autism diagnosis? If yes, how? Yes/No/I do not know

Lived experiences

1. If you are in a situation, which can vary across instances (there will likely be slight variations in what you have previously experienced, for example on a trip to a supermarket or a meal with friends), would you treat it as a new situation, or try to rely on what you would have expected in similar circumstances. If you are able, please give an example of when this happened to you?
2. Imagine you have made friends with someone in a specific context (e.g. at work / at cafes/ at football practice…) and then you meet them somewhere different to where you would usually meet them. Thinking about these two situations – the old one ( where you became friends) and the new one (somewhere different than where you usually meet each other) do you think you would be able to recognise patterns in how your friend behaves across contexts, or would you find your friend's behaviour to be unpredictable from one situation to another?
3. If you have a specific routine for doing things (e.g. morning routine), how would you react if something unexpected happened during this routine (e.g. a phone call)?

1. Let us imagine that in your regular doctor’s office, there is a wall clock that sits on top of the door leading to the bathroom. The next time you visit, the clock has been moved to a table. The time after that, the clock is back on the wall. Next time when you visit, where would you expect the clock to be, if anywhere? Why?
2. a) Do you have a special interest or a hobby that you spend a large amount of time on (i.e. learning everything about different species of birds, computer games, trains etc.)?

5 b) What do you enjoy about that special interest or hobby?

5 c) How does the special interest or hobby make you feel?

1. Do you have any coping mechanisms (e.g. breathing exercises, fiddling with an object, listing all the characters in your favourite movie, smelling a nice smelling object etc....) that you use when you become too overwhelmed? If yes, could you tell us when you use them and how they help you?
2. What do you think are the most important parts of autism that should be captured by a theory trying to explain it? *free
3. A) Are you aware of any theories that attempt to explain autism? If you know any, please tick ‘yes’ regardless of how well you actually know them.

b) Please describe your understanding of them and if you agree with any of them.

Autism Theory

There have been several new theories that have come up in recent years that attempt to provide an explanation of autism as a condition. We would like to present a short description of one specific theory that we are interested in and would like to ask you about your interpretations of it as well how you feel about the theory.

The theory states that:

*From our experiences, we make predictions about situations and their outcomes. The next time we encounter such a situation, we receive input from the environment which either fits or does not fit with our predictions. If the input does not fit with our prediction, we have to judge whether we need to make a new rule for this event. Generally, typically developing individuals are happier to stick with their original prediction and just accept that there will be errors. The theory says that people with autism (or autistic individuals) are more likely to make a new rule for the situation rather than seeing the situation as a variation of what was previously experienced. Situations in our world don’t tend to happen exactly the same way each time. This makes the world a more difficult place to be for people with autism as everything is seen as being new. The theory says that people with autism (or autistic individuals) form predictions / expectations of the world but these predictions / expectations are very specific. Therefore, they are continuously making new predictions for specific situations.*

1. How confident are you in your understanding of this theory? *scale 1-10 (not at all to Very much so
2. Comprehension question: If you had to summarise the above paragraph in your own words, what would you say that the theory is trying to convey about autistic individuals (or individuals with autism).

**Below, we have provided you with some examples of how this theory might interpret some experiences of people with autism**

**Sensory sensitivity:**

"Remi is a school-aged child with an autism diagnosis. One day at school a fire alarm goes off. Remi gets very scared by the fire alarm because it is very loud and unexpected. From that day onwards, Remi does not want to go to school or is consistently anxious about attending school. The theory we described earlier explains this as: The startling and unpleasant experience of the fire alarm has produced large negative emotions in Remi. That event was unexpected as it did not fit with the prediction about a regular day at school. Remi is now terrified of experiencing that same event. Although the fire alarm is only a very rare event, Remi has not separated it from the environment and has learned to expect that same negative experience at school. On the other hand, a neurotypical child would most likely perceive the fire alarm as a coincidence and a rare occurrence."

- 1. How well do you think you understand this example? *sliding scale 1-10 (not at all to Very much so)
  2. Can you think of an example related to your sensory experiences? Please describe it.

**Routines and repetitive behaviours:**

"Alex is school-aged child and has been given an autism diagnosis. One day, Alex is taken by their dad for ice-cream on the way home from school. This was a pleasant experience. From now on Alex insists on going through that route and having ice-cream after school. The theory we described earlier explains this as: Alex incorporated this one-off ice-cream shop visit into their routine of walking home from school. Alex has developed an expectation that after school, they must go and get ice-cream. Now the pleasant experience is not only associated with the ice-cream but also with the walk home from school. Although having ice-cream once they get home would also be pleasant, the routine of walking home from school has been created, thanks to the initial good experience binding the two events together. If they take a different route and do not buy ice cream, Alex will be distressed, as they do not know what might happen on the way home. In a similar situation, a neurotypical child will perceive this ice-cream shop visit as a rare event and will not create the expectation that they have to get ice-cream every time."

- 1. How well do you think you understand this example? *sliding scale 1-10(not at all to Very much so)
  2. Can you think of an example related to your routines? Please describe it.

**Social interactions**:

"Samar is an adult diagnosed with autism. One day, they were in a social situation, which was difficult to navigate. Samar perceived the situation as being completely new. During a later conversation with their partner about it, it became clear that the situation was very similar to a social interaction that they had experienced before. The theory we described earlier explains this as: Social situations are by their own nature a lot more variable than the rest of our lives. Therefore, very rarely would a social situation be identical to one we have already experienced. Samar did not recognise that this situation was similar to an already existing prediction. In comparison, a neurotypical individual would be able to see the interaction as resembling an already existing one, i.e. they would be more lenient with whether the interaction fits with their prediction and would have an easier time navigating it."

- 1. How well do you think you understand this example? *sliding scale 1-10(not at all to Very much so
  2. Can you think of an example related to your interactions with friends? Please describe it.
  3. Can you think of an example related to your interactions with new people you have just met? Please describe it.

**Stimming example:**

“Andy works as a well-being supporter. Sometimes, long working hours cause them to become extremely sensitive to their surroundings. During lunchtime, they are bombarded with different sensory information. Andy senses the different tastes of food and the different sounds all while looking out for service users who might need his assistance. This causes sensory overload which makes them very anxious. To be able to focus, Andy rotates his wrists repeatedly at a rhythmic pace. This allows them to calm down, concentrate on the situation at hand and gain control to perceive their environment less intensely. According to the theory we described earlier, a neurotypical individual only pays attention to the relevant information, filtering out the rest. Andy, on the other hand, attends to all the information equally regardless of how important it is. However, when Andy engages in a repetitive behaviour, Andy creates a predictive environment which helps them filter out irrelevant information and cope with the uncertainties.”

- 1. How well do you think you understand this example? *sliding scale 1-10(not at all to Very much so)
  2. The theory would state that being in an unpredictable world can be stressful and therefore stimming is a way of creating an environment that is very predictable. What do you think about this and can you give us an example?

**General examples**

**Please now think generally across all examples given**

1. Can you think of an incident where an event happened exactly as you had expected? How did it make you feel?
2. Do you rely on previous experiences to help guide you in new situations? (Yes/ No), If you rely on previous experiences do you feel you actively have to recall and compare the present situation to the previous ones, or is it something that happens naturally?
3. Do you think that the theory we described above, in general, fits with your lived experience? Please elaborate with a couple of sentences
4. Can you think of any other examples from your life that we have missed, but fit within the theory we described above?

Do you have any further comments?
